# Spatial Distribution and Molecular Identification of *Leishmania* Species from Endemic Foci of South-Eastern Iran

**Published:** 2012

**Authors:** F Sharifi, I Sharifi, M Zarean, M Hakimi Parizi, MR Aflatoonian, M Fasihi Harandi, R Zahmatkesh, M Mashayekhi, AR Kermanizadeh

**Affiliations:** 1 Leishmaniasis Research Center, Kerman University of Medical Sciences, Kerman, Iran; 2 Dept. of Parasitology, Ahvaz Jundishapur University of Medical Sciences, Ahvaz, Iran; 3 Research Center of Tropical and Infectious Diseases, Kerman University of Medical Sciences, Kerman, Iran; 4 Provincial Health Center, Kerman University of Medical Sciences, Kerman, Iran; 5 District Health Center, Bam Health System, Kerman University of Medical Sciences, Iran

**Keywords:** Cutaneous leishmaniasis, *Leishmania* species, Identification, Nested-PCR, Iran

## Abstract

**Background:**

Cutaneous leishmaniasis constitutes a major public health problem in many parts of the world including Iran. The primary objective of this study was to identify *Leishmania* species in endemic districts of Kerman Province, south-eastern Iran.

**Methods:**

This study was conducted by random sampling as cross- sectional descriptive between 2008 and 2010. Overall, 203 skin scraping smears were taken from the patients. Nested –PCR was performed to amplify variable minicircle fragments of *Leishmania* kDNA.

**Results:**

Bam was the most infected district (71.1%), followed by Kerman (14.7%), Jiroft (5.4%), Baft (2.7%), Sirjan (1.6%), Shahr-e Babak (1.5%) and others (3.0%). *L. tropica* was the most common species identified (194 cases, 95.6%), while *L. major* was found in only 9 cases (4.4%). Of 203 identified patients, all species in Bam (l07 cases), Kerman (32 cases), Jiroft (l6 cases) and Shahr-e- Babak (l1 cases) were detected as *L. tropica*, whereas infected subjects in Baft and Sirjan showed *L. tropica* or *L. major*. Characterization of *Leishmania* species resulted in generation of 750 bp and 560 bp fragments, corresponding to those of *L. tropica* and *L. major*, respectively.

**Conclusion:**

*L. tropica* is the main species (95.6%) caused ACL in endemic areas of Kerman Province; however *L. major* is present in low level (4.4%).

## Introduction

Leishmaniasis remains a public health challenge in 88 countries in tropical and sub- tropical areas with considerable morbidity and mortality (1). Cutaneous leishmaniasis (CL) is a public health and social problem in many countries including Iran. Both epidemiological forms of CL are present in Iran; zoonotic CL (ZCL) caused by *Leishmania major* and anthroponotic CL (ACL) due to *L*. *tropica*. Cutaneous leishmaniasis is endemic in many parts of Kerman Province with increasing incidence and expanding to new foci (2, 3).

The most traditional means of diagnosing CL include direct smear and culture media preparations of *Leishmania* amastigote and promastigote stages, respectively. Both methods are certainly of value, although the culture method is time consuming. These procedures offer the advantage of simplicity, but they do not discriminate the organism in species level (4, 5) and often with variable and low sensitivity (5–7). However, reliance on clinical ground and epidemiological features is due to necessity and there are several reasons why specific identification should be used as an essential part of the control strategy against leishmaniasis (8–10).

At present, there is no vaccine available against any form of leishmaniasis. Because recovery from infection is usually associated by a strong immunity; hope for the development of a vaccine for human has been high (11). The current treatment of choice, mainly antimonial compounds, have considerable side–effects and hence should not be given without justification (12). Polymerase chain reaction (PCR) is a reliable technique with greater sensitivity than routine methods or clinical and epidemiological characteristics (5–7, 9).

The main objective of this study was to identify the causative *Leishmania* species involved in different endemic districts within Kerman Province, southeastern Iran. Knowledge of CL identification is very important, since species differentiation is instrumental in selection of optimal therapy and treatment regimens. Moreover, this method can be carried out in surveys for detection of cases, reservoir hosts and vectors for better understanding of the transmission mechanisms and control of this complex disease (13).

To our Knowledge, this is the first comprehensive study in terms of geographical distribution and molecular identification of *Leishmania* species in Kerman Province, south – eastern Iran.

## Materials and Methods

### Study area

**Fig. 1:**
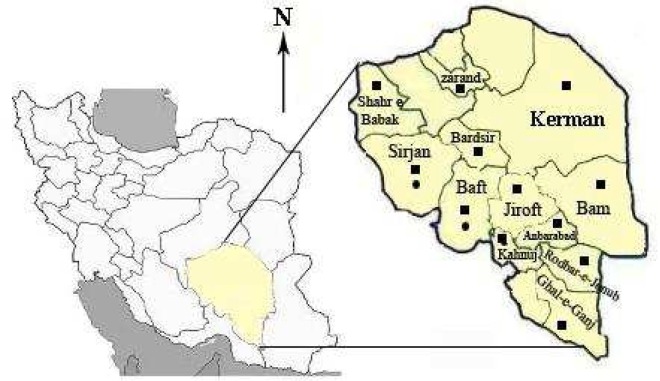
Special distribution of *Leishmania tropica* (■) and *Leishmania major* (●) in endemic districts, Kerman Province, south-eastern Iran

The samples were taken from the subjects in CL endemic districts within the Province of Kerman, with a population of 2,700,000. This province covers an area of 181714 km^2^ and is located in the south east of Iran (Fig. 1). It is the largest province and includes 11% of the total area of the country. The main districts are sixteen, but CL is endemic in six districts (Bam, Kerman, Jiroft, Shahr- e- Babak, Baft and Sirjan) and sporadic cases are reported to a lower extent from other districts. The climate varies in different regions depending on the relief of the land. The north, northwest and central areas (Kerman, Shahr- e- Babak, Baft and Sirjan) experience a dry and moderate climate, whereas in the south and southeast (Bam and Jiroft) the weather is warm and relatively humid.

### Data collection and sampling

This study was conducted by random sampling as cross-sectional descriptive in two series. First, prior to identification of *Leishmania* species, the present status of CL was assessed. A base line survey was carried out in a retrospective manner for two years, from March 2008 to February 2009. All the CL patients registered and confirmed by direct smear preparation were included. Second, prospective data on CL and demographic characteristics were collected in similar manner over a period of sixteen months, from March 2009 to July 2010.

Since the CL is a reportable disease within the Province of Kerman and control programs are integrated into Primary Health Care (PHC) system, the proportions of samples from endemic localities within districts were relatively based on the annual reports by the Kerman Provincial Health Center. Therefore, 203 samples were taken from the patients referred to the health clinics of the districts. CL patients with a history of travelling to other endemic areas within a period of one year prior to sampling were excluded. Clinical and demographic characteristics of the infected individuals including sex, age, location and number of lesions were assessed. No samples were been taken from Rafsanjan district due to administrative segregation of health facilities from Kerman Province.

### Sample preparation

Skin scrapings were taken from the periphery of active lesions, smeared on a glass slide, fixed with methanol, stained by Giemsa and observed under a light microscope for presence of amastigote stages (Leishman bodies).

### DNA extraction

DNA was prepared from direct smears. Smear scrapings were transferred to 1.5- ml micro tubes and centrifuged three times in physiological saline solution. DNA was extracted by proteinase K using the High Pure PCR Template Purification Kit (Roche, Germany), according to the manufacturer's instructions.

### Nested – PCR

Nested – PCR was performed as follows: in the first stage two external primers CSB1XR (CGAGTAGCAGAAACTCCCGTTCA) and CSB2XF(ATTTTTCGCGATTTTCGCAGAACG) and in the second step, two internal specific primers 13Z (ACTGGGGGTTGGTGTAAAATAG) and LiR (TCGCAGAACGCCCCT) were used for amplification of variable minicircles of *Leishmania* kDNA.

The PCR products were visualized by 1.5% agarose gel electrophoresis (Uvitech, Cambridge UK), using a 100- bp DNA ladder marker at 260 nm wavelength. A negative and a positive control including *L. major* (MRHO/ 64/ Nadim -1 strain) and *L. tropica* (MHOM/Sudan/58/OD strain) were used in each round of PCR and electrophoresis. *L. tropica* and *L. major* provided fragments of 750 bp and 560 bp, respectively.

### Ethics and analysis

Oral consent of the subjects was obtained. All the CL patients were referred to receive proper medication free of charge. Data were entered into a computer, using a SPSS software and χ^2^– test to determine any significant difference between disease distribution and demographic characteristics at *P*<0.05.

## Results

Overall, mean annual CL was 2512 cases for 2008 and 2009. Bam was the most infected district (71.1%), followed by Kerman (14.7%), Jiroft (5.4%), Baft (2.7%), Sirjan (1.6%), Shahr-e Babak (1.5%), Anbarabad (1.1%), Rodbar-e-Jonoob (1.0%), Ghal-e-Ganj(0.3%), Kahnouj, Zarand and Bardsir 0.2% each (Table 1). *L*. *tropica* was the most common species identified by nested-PCR in Kerman Province (194 cases, 95.6%), while *L. major* was found in only 9 cases (4.4%). Of 203 identified patients, all species in Bam (107 cases), Kerman (32 cases), Jiroft (16 cases) and Shahr-e- Babak (11 cases) Anbarabad (4 cases), Rodbar-e-Jonoob (3 cases), Ghal-e-Ganj, Kahnouj, Zarand and Bardsir one case each, were detected as *L.tropica*, whereas infected subjects in Baft and Sirjan showed either species, (5.1% and 3.6% *L. tropica* and 33.3% and 66.7% *L. major*, respectively).

**Table 1:** Spatial distribution and molecular identification of *Leishmania* species, Kerman Province, south-eastern Iran by district

District	Mean annual cases	Nested-PCR	

		*L. tropica*	*L. major*
	No. (%)	No. (%)	No. (%)
**Bam**	1787(71.1)	107(55.2)	0(0)
**Kerman**	369(14.7)	32(16.5)	0(0)
**Jiroft**	136(5.4)	16(8.2)	0(0)
**Baft**	67(2.7)	10(5.1)	3(33.3)
**Sirjan**	41(1.6)	7(3.6)	6(66.7)
**Shahr-e-Babak**	38(1.5)	11(5.7)	0(0)
**Anbarabad**	27(1.1)	4(2.1)	0(0)
**Rodbar-e-Jonoob**	24(1.0)	3(1.6)	0(0)
**Ghal-e-Ganj**	7(0.3)	1(0.5)	0(0)
**Kahnouj**	6(0.2)	1(0.5)	0(0)
**Zarand**	5(0.2)	1(0.5)	0(0)
**Bardsir**	5(0.2)	1(0.5)	0(0)
**Total**	2512(100.0)	194(100.0)	9(100.0)

*L. tropica* and *L. major* were equally distributed among female and male genders, with no significant difference (Table 2). The mean age was 27.5 years±SD 8.1 (range, 5–75 years). All age groups were infected; although the rate of CL lesions in age group > 41 years was 18.7% which was significantly lower than 21–40 years or < 20 years age group (*P*<0.05).

**Table 2:** Molecular identification of *Leishmania* species, Kerman Province, south-eastern Iran by sex

Sex	Female	Male	Total

	No. (%)	No. (%)	No. (%)
***L. tropica***	102(95.3)	92(95.8)	194(95.6)
***L. major***	5(4.7)	4(4.2)	9(4.4)
**Total**	107(100.0)	96(100.0)	203(100.0)

**Fig. 2:**
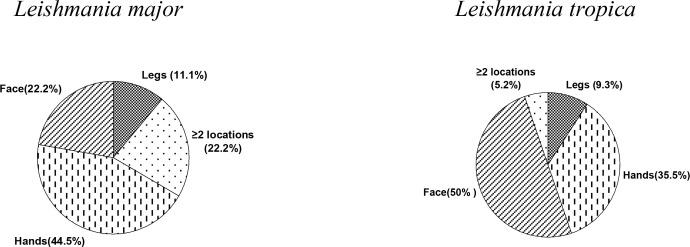
Molecular identification of *Leishmania* species, Kerman Province, south-eastern Iran by location of lesions

Distribution of *Leishmania* species by location of lesions are shown in Fig. 2. For CL due to *L. tropica*, lesions were mainly located on the face (50%), followed by hands (35.5%), legs (9.3%) and the remaining (5.2%) on two or more locations. In contrast, in patients with *L. major*, lesions were mostly on the hands (44.5%), equally located on the face or two or more places (22.2% each) and finally legs (11.1%). The mean number of lesions was 1.5 for *L. tropica* and 2.8 for *L. major*, indicating a significant difference among the two species (*P*<0.01).

**Fig. 3:**
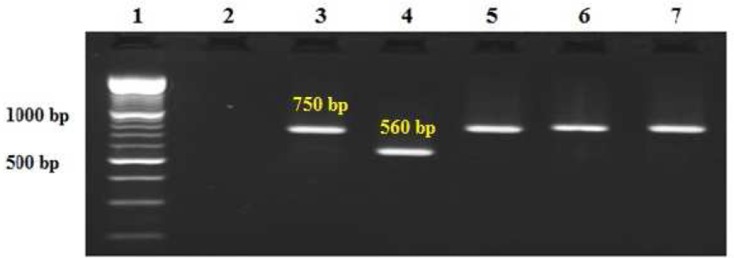
Agarose gel electrophoresis of *Leishmania* isolates. Lane 1, DNA size marker 100bp; lane 2, negative control; lane3, *L. tropica* (positive control 750bp); lane 4, *L. major* (positive control 560 bp); lanes 5, 6, 7 *L. tropica* isolates obtained from skin lesions of the patients, Kerman Province, south-eastern Iran

Further characterization of the causative parasite species by nested-PCR, using kinetoplast minicircle fraction amplification of 194 cases (95.6%), resulted in the generation of a 750 bp DNA and 9 patients (4.4%) displayed a fragment of 560 bp, corresponding to those of *L. tropica* and *L. major*, respectively (Fig. 3).

## Discussion

Cutaneous leishmaniasis constitutes a major public health problem in Iran and worldwide (1). Identification of different species of CL is important for treatment modality and control strategies (8, 12, 14). Most of the previous differentiations were based on extrinsic factors such as clinical, epidemiological and biological characteristics of host – parasite features. Due to lack of strict vector-parasite specificity in several localities and various clinical manifestations and different epidemiological forms, the above criteria are insufficient and constitute major limitations for definitive identifications of the causative parasite (4, 5).

At present a variety of biochemical, immunological and molecular methods, based on intrinsic factors, have been developed for precise identification of *Leishmania* species. Among these, currently, the most commonly used method is DNA- based techniques, using PCR and specific primers for species and even strains characterization. The PCR analysis of *Leishmania* species is a precise and powerful method, which has contributed for many years to studies in leishmaniasis. In addition to high sensitivity and specificity, this method has also been used for taxonomic differentiation of *Leishmania* species (5, 6, 7, 10, 15).

In ACL endemic foci within Kerman Province where clinical forms such as leishmaniasis recidivans (lupoid form) is a common phenomenon, PCR method could be of prime importance in specific detection of the causative agent. The scarcity of amastigotes in the lesions in direct smears and tissue specimens, otherwise easily leads to misdiagnosis (16).

*L. tropica* was the most common species identified in this study in endemic foci of Kerman Province mainly in Bam, Kerman, Jiroft,Shar-e- Babak Anbarabad, Rodbar-e-Jonoob, Ghal-e-Ganj, Kahnouj, Zarand and Bardsir. The predominance of ACL due to *L. tropica* has already been confirmed mainly in Bam in several studies (4, 17, 18). However, in Baft and Sirjan, both species are present, but *L. major* is in higher proportion as compared to *L. tropica*. *L. major* has previously been reported to be the sole causative agent of a ZCL epidemic in the southern areas of Baft district (2). Our results are consistent with Hajjaran et al. (19) in Mashhad, where 94.2% of isolates were *L. tropica*, while contrary to those reported by Maraghi et al. (6) who indicated 90% of isolates in Shush, Khuzestan Province, Iran were *L*. *major*. On the other hand, majority of the provinces of Iran are mixed with *L. tropica* and *L. major* (9, 20).

In this study, the individuals > 41 years showed a significantly lower CL infection than other groups. The reason for such difference is probably due to low exposure of >41 years as compared to the other two age groups. CL was prevalent in both sexes, similarly. The result is consistent with previous findings from Bam (3, 4).

The CL characteristics of *L. tropica* and *L. major* cases in terms of the location of lesions and the mean number of sores are consistent with those of ACL and ZCL previously reported from Bam (18), Baft (2) or around the country (5, 7, 9, 21, 22) or globally (23).

The finding of this study showed that *L. tropica* was the main species (95.6%) caused ACL in endemic areas of Kerman Province, however *L. major* is present in low level (4.4%). Nested –PCR could be a valuable tool in selection of optimal therapy and treatment regimens, especially in complex localities where more than one *Leishmania* species is present. This method is also important for strategic planning and future control programs.
